# Editorial: The Metabolic-Inflammatory Axis in Brain Aging and Neurodegeneration

**DOI:** 10.3389/fnagi.2017.00209

**Published:** 2017-06-28

**Authors:** Fei Yin, Jia Yao, Roberta D. Brinton, Enrique Cadenas

**Affiliations:** ^1^Department of Pharmacology and Pharmaceutical Sciences, School of Pharmacy, University of Southern CaliforniaLos Angeles, CA, United States; ^2^Center for Innovation in Brain Science, University of ArizonaTucson, AZ, United States; ^3^Department of Pharmacology, College of Medicine, University of ArizonaTucson, AZ, United States

**Keywords:** brain metabolism, redox homeostasis, Alzheimer's disease, neuroinflammation, mitochondria

## Introduction

Impairment of energy metabolism is a hallmark of brain aging and several neurodegenerative diseases, such as the Alzheimer's disease (AD). Age- and disease-related hypometabolism is commonly associated with oxidative stress and they are both regarded as major contributors to the decline in synaptic plasticity and cognition. Neuroinflammatory changes, entailing microglial activation and elevated expression of inflammatory cytokines, also correlate with age-related cognitive decline. It is still under debate whether the mitochondrial dysfunction-induced metabolic deficits or the microglia activation-mediated neuroinflammation is the initiator of the cognitive changes in aging and AD. Nevertheless, multiple lines of evidence support the notion that mitochondrial dysfunction and chronic inflammation exacerbate each other, and these mechanistic diversities have cellular redox dysregulation as a common denominator.

This research topic focuses on the role of a metabolic-inflammatory axis (Yin et al., 2016) encompassing the bioenergetic activity, brain inflammatory responses, and their redox regulation in healthy brain aging and neurodegenerative diseases. Dynamic interactions among these systems are reviewed in terms of their causative or in-tandem occurrence and how the systemic environment,—e.g., insulin resistance, diabetes, and systemic inflammation—, impacts on brain function.

## Energy metabolism in brain aging and neurodegeneration

Brain energy metabolism starts with the uptake of energy fuels—primarily glucose—from the cerebrovasculature. In the review contributed by Lourenço et al., the derailment of neurovascular and neurometabolic functions was discussed in both normal aging and AD, with the nitric oxide (·NO)-mediated neurovascular coupling being proposed as a key factor implicated in the progression of neuronal dysfunction.

In addition to glycolysis, glucose in the brain is metabolized via the pentose phosphate pathway (PPP) to generate NADPH—the major reducing equivalent in the cell supporting GSH- and thioredoxin-dependent antioxidant systems (Yin et al., 2014). Bouzier-Sore and Bolaños suggested that age- and disease-related decline in glucose uptake is responsible for not only the reduced bioenergetic capacity but also the neuronal oxidative damage, inasmuch as the contribution of PPP to neuronal glucose metabolism is underestimated with current assessment approach.

Following their uptake into the brain and subsequent cytosolic reactions, energy fuels are primarily metabolized in mitochondria—the only organelles that possess their own genome (mtDNA). Toward this unique property, Wang and Brinton comprehensively discussed the advances in the field in support of mitochondrial genetic variance/haplotypes as another important genetic risk factor for AD, together with APOE4 and chromosomal sex. Moreover, a precision medicine approach was proposed to incorporate these major risk factors into the design and development of future therapeutics for late onset AD.

Regulation of mitochondrial function is coordinated by both mtDNA and the nuclear genome, with the latter encoding ~99% of the proteins of these organelles. To maintain an efficient, dynamic, and responsive mitochondrial network, a complex multi-layer quality control system is utilized by the cell (Yin and Cadenas, 2015). Recently, the field of mitochondria biology has been focused on the dynamic and interactive features of these semi-autonomous organelles and a complex multi-layer quality control system coordinating their function with environmental changes. In this regard, Zorzano and Claret reviewed the vital role of mitochondrial dynamics in the pathogenesis of neurodegenerative diseases, as well as in the hypothalamic dysfunction that is implicated in imbalanced energy metabolism.

## Redox regulation connects brain metabolism and neuroinflammation

The aging- and neurodegenerative brains are found to be associated with a mild, chronic neuroinflammation primarily due to dysregulated innate immunity, with microglia senescence playing a central role. In the review by von Bernhardi et al., the key elements and pathways of microglia action and their potential as a therapeutic target in brain aging and AD were discussed comprehensively. It was proposed that microglia aging and its mal-activation, induced by mitochondrion- and non-mitochondrion-derived oxidative stress and endolysosomal dysfunction, initiate and further exacerbate neuroinflammation, resulting in synaptic dysfunction, and eventually neurodegeneration.

It is well-known that deficits in metabolic and mitochondrial function lead to the impairment of redox homeostasis, which is one of the major stimulators of the neuroinflammatory responses in aging and AD. The review by Gamba et al. suggested that altered cholesterol metabolism (and hypercholesterolemia) mediates such a connection and thereby contributes to AD progression, via a vicious cycle involving redox perturbation, oxidized cholesterol, neuroinflammation, Aβ load, and neuronal damage.

Further, in the review contributed by De Felice and Lourenco, brain metabolic dysregulation in AD was expanded from glucose metabolism to Aβ oligomer (AβO)-induced neuroinflammation and ER stress (unfolded protein response, UPR), with the latter two processes impacting not only synaptic and cognitive functions, but also peripheral insulin resistance, glucose intolerance, and diabetes. It was also suggested that UPR activation might be a common pathogenic mechanism shared by multiple neurological disorders.

## Peripheral- and endocrine interactions with the brain metabolic-inflammatory axis

The connection between brain function and the systemic environment is bidirectional, as indicated by the important role of peripheral metabolism in the progression of brain aging and neurodegeneration. In this Research Topic, Christensen and Pike discussed the role of obesity and systemic inflammation in AD pathogenesis with a special emphasis on women, who have a two-fold increased AD risk compared to men. In addition to the obesity-induced inflammation, increased AD risk in females also correlated with the elevated inflammation due to estrogen depletion upon perimenopause and menopause. The authors thereby proposed an interactive set of risk factors implicated in AD, including aging, menopause, adiposity, and inflammation.

Like adiposity, the risk of type 2 diabetes (T2D) for AD was also mediated through low-grade inflammation. In the report by Gorska-Ciebiada et al. in a group of T2D patients, it was found that serum levels of inflammatory parameters, such as C-reactive protein, advanced glycation end products (AGE), and their receptors, were increased only in those with mild cognitive impairment (MCI), a critical transitional stage between normal cognitive aging and AD.

Brain function and neurodegenerative risks are not only affected by the systemic metabolic status, but also the endocrine system. As reported by Morgan and Finch, the onset of perimenopause increased the estrogen receptor-alpha to estrogen receptor-beta ratio (ERα/ERβ) in astrocytes, which was associated with impaired neurotrophic responses to estradiol. These endocrine-related changes occurred during female perimenopausal transition could thus be implicated in the increased risk for AD in women (Brinton et al., 2015).

The review contributed by Paul et al., focused on the role of glucocorticoids—another family of anti-inflammatory steroid hormones—and their receptors in the context of stress-induced cognitive decline in aging and AD. The interactive molecular links of glucocorticoids with the cholinergic system and the hypothalamic-pituitary-adrenal (HPA) axis were proposed as a contributing factor to cognitive impairment and a target of pharmacotherapeutic strategies.

Two potential neuroprotective strategies targeting the metabolic or inflammatory pathways were discussed in this topic. In a study utilizing an early stage Parkinson's disease animal model, Farmer et al. suggested that immune system signaling molecules, granulocyte macrophage-colony stimulating factor (GM-CSF) and erythropoietin (EPO), exhibited neurotrophic and neuroprotective capabilities to re-innervate striatal neurons. In terms of nutraceutical strategies targeting brain oxidative and inflammatory pathways, Ong et al. contributed a literature review of the neuroprotective mechanisms and potential of ginsenosides, the bioactive ingredients in ginseng root, against multiple neurodegenerative disorders.

## Perspectives

Brain aging and neurodegenerative diseases have a multifactorial nature. For example, AD is characterized by not only the accumulation of the Aβ oligomers and fibrils, but also metabolic and inflammation changes such as glucose hypometabolism, blood-brain-barrier (BBB) disruption and glial activation. Recent failures of multiple clinical trials targeting Aβ continue to fuel the debate on the Aβ-centric view of AD pathogenesis. We hope this research topic has created a forum for in-depth discussions that help evolve the sequential view of the mechanisms inherent in brain aging and progression of neurodegeneration to the understanding that several mechanisms—centered on a dynamically coordinated mitochondrial network and the metabolic-inflammatory axis—co-exist and interact with each other with significant contribution from the systemic environment such as insulin resistance and peripheral inflammation.

## Author contributions

FY drafted the manuscript that was reviewed and edited by JY, RDB, and EC. All authors co-edited the Research Topic.

### Conflict of interest statement

The authors declare that the research was conducted in the absence of any commercial or financial relationships that could be construed as a potential conflict of interest.
